# DDOST Correlated with Malignancies and Immune Microenvironment in Gliomas

**DOI:** 10.3389/fimmu.2022.917014

**Published:** 2022-06-23

**Authors:** Xiaojing Chang, Jie Pan, Ruoyu Zhao, Tianfang Yan, Xinrui Wang, Cunle Guo, Yining Yang, Guohui Wang

**Affiliations:** ^1^ Department of Radiotherapy, the Second Hospital of Hebei Medical University, Shijiazhuang, China; ^2^ Department of Pathology, Stanford University School of Medicine, Stanford, CA, United States; ^3^ Department of Radiotherapy, Tianjin First Center Hospital, Tianjin, China; ^4^ Department of Neurological Diagnosis and Restoration, Osaka University Graduate School of Medicine, Suita, Japan

**Keywords:** DDOST, glioma, progression, microenvironment, prognosis

## Abstract

Among the most common types of brain tumor, gliomas are the most aggressive and have the poorest prognosis. Dolichyl-diphosphooligosaccharide protein glycosyltransferase non-catalytic subunit (DDOST) encodes a component of the oligosaccharide transferase complex and is related to the N-glycosylation of proteins. The role of DDOST in gliomas, however, is not yet known. First, we performed a pan cancer analysis of DDOST in the TCGA cohort. The expression of DDOST was compared between glioma and normal brain tissues in the GEO and Chinese Glioma Genome Atlas (CGGA) databases. In order to explore the role of DDOST in glioma, we analyze the impact of DDOST on the prognosis of glioma patients, with the CGGA 325 dataset as a test set and the CGGA 693 dataset as a validation set. Immunohistochemistry was performed on tissue microarrays to examine whether DDOST has an impact on glioma patient survival. Next, using single-cell sequencing analysis, GSEA, immune infiltration analysis, and mutation analysis, we explored how DDOST affected the glioma tumor microenvironment. Finally, we evaluated the clinical significance of DDOST for glioma treatment by constructing nomograms and decision curve analysis (DCA) curves. We found that DDOST was overexpressed in patients with high grade, IDH wild type, 1p19q non-codel and MGMT un-methylated, which was associated with poor prognosis. Patients with high levels of DDOST, regardless of their clinical characteristics, had a worse prognosis. Immunohistochemical analysis confirmed the results of the above bioinformatics analysis. Mechanistic analysis revealed that DDOST was closely associated with the glioma microenvironment and negatively related to tumor-infiltrating B cells and CD4+ T cells and positively related to CAFs and tumor-associated macrophages. In conclusion, these findings suggested that DDOST mediated the immunosuppressive microenvironment of gliomas and could be an important biomarker in diagnosing and treating gliomas.

## Introduction

Gliomas are the most common malignant primary intracranial tumors that arise from glial or precursor cells, accounting for approximately 24.5% of all primary brain and other central nervous system (CNS) tumors and 80.9% of CNS malignancies (1). According to the 2021 fifth edition World Health Organization (WHO) classification, gliomas are categorized into four grades, WHO 1–4 (2). WHO 1 and WHO 2, with a relatively good OS, are defined as low-grade gliomas, and WHO 3 and WHO 4, which are related to a poor prognosis, are defined as high-grade gliomas. The 5-year survival rate has a large variation from 94.7% for pilocytic astrocytoma (WHO 1 grade) to 6.8% for glioblastoma (WHO 4 grade) (1). More recently, molecular biomarkers, such as isocitrate dehydrogenase (IDH), telomerase reverse transcriptase (TERT), and O-6-methylguanine-DNA methyltransferase (MGMT), have gained importance in providing diagnostic, prognostic, and therapeutic information (3). The leading change in the 2021 fifth edition WHO classification is advancing the role of molecular diagnostics, which could provide the most accurate classification of CNS neoplasms and guide clinical management and prognosis of gliomas (2). Thus, the investigation of biomarkers is a pivotal area in the research of gliomas.

Glycosylation, one of the important posttranslational modifications of proteins, is involved in various cell biological processes, and is closely related to many pathological processes, such as tumorigenesis and inflammatory responses (4). Glycosylation modifications are classified into O-glycosylation, N-glycosylation, C-glycosylation, and glycosylphosphatidylinositol anchor linkage based on the glycosylation site, among which O-glycosylation and N-glycosylation are the most common. Alterations in glycosylation could produce tumor-associated polysaccharides or glycoproteins that can serve as tumor-related markers and correlate with tumor development and prognosis (5). Dolichyl-diphosphooligosaccharide protein glycosyltransferase non-catalytic subunit (DDOST) encodes a component of the oligosaccharide transferase (OST) complex and is related to the N-glycosylation of proteins. Some researchers found that patients with skin squamous cell carcinoma with highly expressed DDOST showed a worse prognosis (6). Moreover, studies have shown that DDOST is an independent prognostic factor in patients with liver cancer, and its expression is positively correlated with the level of Th2 cell infiltration, but negatively correlated with the level of cytotoxic cell infiltration (7). However, no studies have been conducted on DDOST in gliomas.

In this project, we investigated the expression and significance of DDOST in gliomas by bioinformatics and immunohistochemical staining, and preliminarily discussed its impact on the glioma microenvironment. Furthermore, these results were verified by different databases.

## Materials and Methods

### Data Acquisition

Pan cancer analysis data, TCGA Pan Cancer, were obtained from University of California, Santa Cruz (UCSC) (https://xenabrowser.net/). Microarray data of glioma expression profile, GSE4290 (8) and GSE50161 (9), were downloaded from the GEO database. From the TCGA database, gene expression and clinical information files of LGG and GBM were downloaded. Additionally, we analyzed the Chinese Glioma Genome Atlas (CGGA) to obtain RNA profiles and clinical features of gliomas and single-cell sequence information (10). All gene expression data were standardized and batch-processed using the limma R package. In the analyzed dataset, we retained only the gliomas with complete clinicopathological data and survival data, excluding patients with unknown or incomplete data. Single-cell sequencing data were normalized and quality controlled by the Seurat R package.

### Pan Cancer Analysis

We performed differential analysis of DDOST on the pan cancer expression profile data downloaded from UCSC by Wilcoxon rank sum and signed rank tests. Then, the data were subjected to Cox proportional hazards regression models to determine the effects of DDOST on overall survival (OS).

### DDOST Differential Expression in Glioma and Normal Brain Tissue

Firstly, we compared the expression of DDOST in gliomas and normal brain tissues by Wilcoxon rank sum test in CGGA, GSE4290, GSE50161, TCGA, and GTEx databases. Then, the CGGA 325 cohort was used as the test set and the CGGA 693 cohort was used as the validation set. We compared the expression of DDOST in gliomas with different clinical characteristics, such as age (age<42, age≥42), gender (female, male), grade (WHO 2, WHO 3, and WHO 4), IDH (mutant, wild type), 1p19q (codel, non-codel), and MGMT (methylated, un-methylated) status.

### Prognostic Analysis

Kaplan–Meier survival analysis was used to determine the survival prognosis for gliomas. Patients were grouped into the “high” or “low” group based on the median value of DDOST expression. According to the above clinical characteristics, we analyzed the survival subgroups of glioma patients, including different age, gender, grade, and molecular characteristics. The CGGA 325 cohort was used as the test set and the CGGA 693 cohort was used as the validation set. The results were displayed by hazard ratio (HR), 95% confidence intervals (95% CI), and log-rank *p*-value (significant threshold <0.05).

### Tissue Microarray and Immunohistochemistry

Shanghai Outdo Biotech Co., Ltd. (China) provided the tissue microarray applied for this research. The detailed procedure and scoring criteria of immunohistochemistry were referred to previously published articles from our team (11). A mouse monoclonal anti-DDOST antibody at a dilution of 1:100 was purchased from Santa Cruz (sc-74408). This semi-quantitative analysis was done by two independent assessors without prior knowledge of the patient outcome.

### Single-Cell Analysis

Gene alteration information could be obtained from single-cell data in a more detailed way. The processed single-cell data containing 6,148 cells were downloaded from the CGGA database (12). The Seurat R software package was performed to reduce the dimensions of cells and generate t-SNE diagram for cell-type visualization. In order to study the role of DDOST in tumors, we compared the expression differences of DDOST between different cell types.

### Database for Annotation, Visualization, and Integrated Discovery and Gene Set Enrichment Analysis

Differentially expressed genes (DEGs) between gliomas with high vs. low DDOST expression in the CGGA 325 cohort were identified using the limma package on R using |logFC0| > 1.5 and FDR < 0.05 as cutoffs. To further explore its possible mechanism, we conducted GO and KEGG pathway analysis using DAVID 6.8 (https://david.abcc.ncifcrf.gov/) (13). To further explore the possible mechanism, the GSEA 4.0.2 software was used (14). A normalized enrichment score (NES) >1 and false discovery rate <0.05 were considered significant.

### Evaluation of the Effect of DDOST on the Glioma Microenvironment

The fraction of immunocytes in gliomas was estimated using single-sample gene set enrichment analysis (ssGSEA) and CIBERSORT as previously published (11). Mass cytometry data from immune cells can be analyzed in a standardized manner using the Estimate the Proportion of Immune and Cancer cells (EPIC) web-based analytical and discovery platform (15).

### Gliomas Were Clustered According to Immune-Related Genes by NMF

First, we download the list of immune-related genes from the ImmPort database (16). Non-negative matrix factorization (NMF) is an effective dimensionality reduction method that is widely used for molecular pattern recognition of high-dimensional genomic data and provides a powerful approach for class discovery. We extracted immune-related gene expression quantities from the CGGA 325 database and then performed NMF clustering analysis. The optimal number of clusters was calculated according to the values obtained from cophenetic.

### Molecular Characteristic Specific for Low and High DDOST Groups

The TCGA database was used to collect single-nucleotide variants (SNVs) of LGG and GBM. Based on the expression values of DDOST, gliomas were divided into high and low groups. Wilcoxon rank sum test was used to compare gene mutations between the two patient groups. Waterfall plots were utilized to demonstrate the mutation type and mutation expression relationship with DDOST for the top 15 mutations.

### Construction of a Predictive Nomogram

Nomograms referred to quantitative analysis plots representing the functional relationship between multiple variables with a cluster of mutually disjoint segments in plane coordinates. Clinical features such as age, gender, grade, and molecular characteristic of IDH, 1p19q, MGMT, and DDOST were used to build a nomogram. The probability of 1-, 3-, and 5-year OS of patients with glioma was determined using the rms R package. Decision curve analysis (DCA) curves assessed the value of DDOST for prognostic assessment.

## Results

### Characteristics of Patients With Gliomas

We obtained gene expression data from 1,572 gliomas and 41 normal brain tissues by screening publicly available databases, as well as a single-cell sequencing dataset comprising 6,148 cells. Tissue microarray included 121 cases of glioma and 3 cases of normal brain tissue. The CGGA 325 cohort served as the test set and the CGGA 693 cohort served as the validation set. In **Table 1**, the clinical characteristics and molecular characteristics are listed.

**Table 1:** Clinicopathological characteristics of glioma patients from the GEO, TCGA, and CGGA database and tissue microarray.

	GSE4290 (*n* = 176)	GSE50161 (*n* = 130)	TCGA (*n* = 592)	CGGA 325 (*n* = 286)	CGGA 693 (*n* = 429)	Tissue Microarray (*n* = 124)
Age						
<42	NA	NA	241	124	197	44
≥42	NA	NA	346	162	232	77
Gender						
Female	NA	NA	246	110	191	35
Male	NA	NA	341	176	238	86
Normal Tissue	23	13	5	NA	NA	3
Tumor Tissue	153	117	587	286	429	121
Grade						
II	45	NA	211	86	100	37
III	31	NA	234	68	164	40
IV	77	NA	142	132	165	47
IDH Status						
Wild type	NA	NA	219	136	193	NA
Mutation	NA	NA	368	150	236	NA
1p/19q						
Codel	NA	NA	149	57	90	NA
Non-codel	NA	NA	438	229	339	NA
MGMT						
Methylated	NA	NA	NA	147	251	NA
Un-methylated	NA	NA	NA	139	178	NA
Status						
Dead	NA	NA	173	202	278	47
Alive	NA	NA	414	84	151	77

NA, Not available.

### Pan Cancer Analysis Revealed That DDOST Was Highly Expressed in a Subset of Tumors and Associated With Poor Prognosis

**Figure 1:**
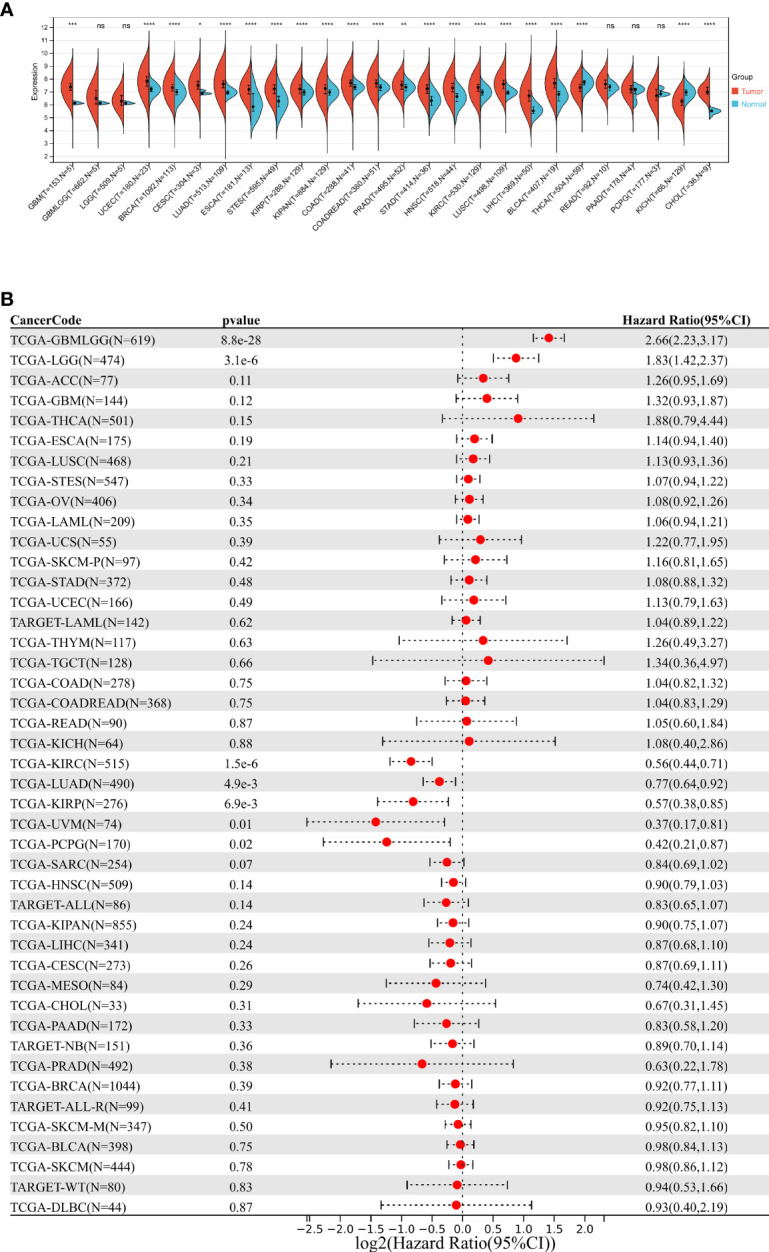
Pan cancer analysis of DDOST expression. **(A)** Analysis of DDOST expression in 33 tumors on TCGA. *, **, ***, **** indicate *p* < 0.05, *p* < 0.01, *p* < 0.001, and *p* < 0.0001, respectively; ns, not significant (Wilcoxon test). **(B)** Risk plot of correlation between DDOST levels and OS.

We calculated the expression difference between normal and tumor samples in each tumor *via* the R software, using unpaired Wilcoxon rank sum and signed rank tests for differential significance analysis. Results showed that significant upregulation of DDOST was observed in 19 tumors, such as GBM (tumor: 7.37 ± 0.46, normal: 6.15 ± 0.07, *p* = 2.0e-4) and CESC (tumor: 7.51 ± 0.53, normal: 6.95 ± 0.13, *p* = 0.03), while DDOST was downregulated in only 2 tumors, namely, THCA (tumor: 7.32 ± 0.47, normal: 7.69 ± 0.36, *p* = 3.6e-11) and KICH (tumor: 6.18 ± 0.56, normal: 6.97 ± 0.31, *p* = 2.1e-21) (**Figure 1A**). Cox proportional hazards regression models were performed to analyze the prognostic relationship between gene expression and prognosis within each tumor. High expression of DDOST was associated with poor prognosis in 11 tumor types (**Figure 1B**).

### Gliomas With High DDOST Expression Showed Poor Prognosis

**Figure 2:**
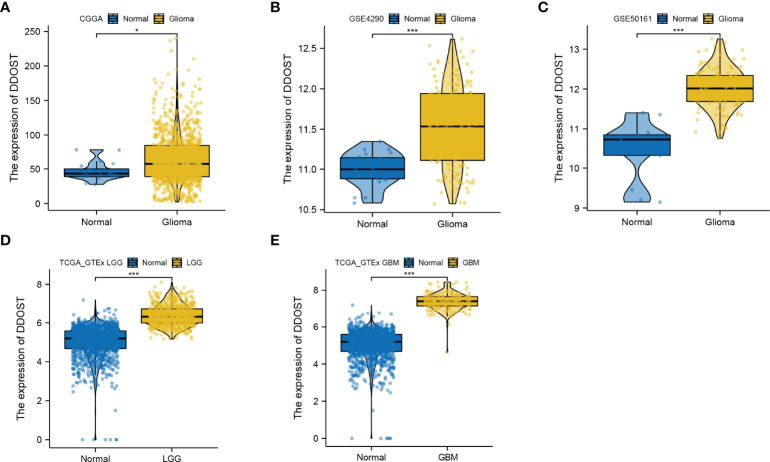
Expression difference of DDOST between glioma and normal tissue. **(A)** The expression of DDOST was different between glioma and normal brain tissue in the CGGA cohort. **(B, C)** In the GSE4290 and GSE50161 cohort, DDOST expression was significantly higher in glioma patients than in normal brain. **(D, E)** In the TCGA and GTEx, the expression of DDOST in LGG and GBM was significantly higher than that in normal tissue. *p < 0.05, ***p < 0.001.

**Figure 3:**
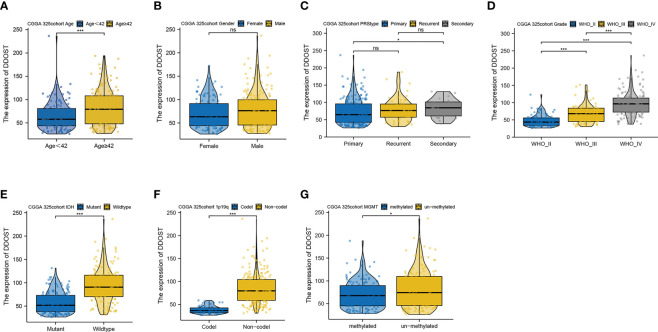
Expression difference of DDOST between different clinical characters in patients with glioma in the CGGA 325 cohort. The expression of DDOST in different age **(A)**, gender **(B)**, PRS type **(C)**, grade **(D)**, IDH **(E)**, 1p19q **(F)**, and MGMT status **(G)**. *p < 0.05, ***p < 0.001. ns, not significant.

**Figure 4:**
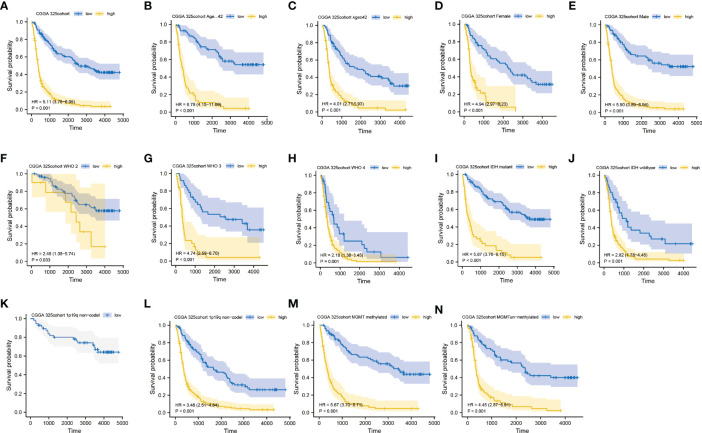
Prediction of outcome of the DDOST in stratified patients in the CGGA 325 dataset. Survival curve was used to analyze OS in the low- and high-DDOST groups in CGGA 325 set **(A)**. Survival analysis of the signature in patients stratified by age **(B, C)**, gender **(D, E)**, grade **(F, H)**, IDH **(I, J)**, 1p19q status **(K, L)**, and MGMT promoter **(M, N)**.

**Figure 5:**
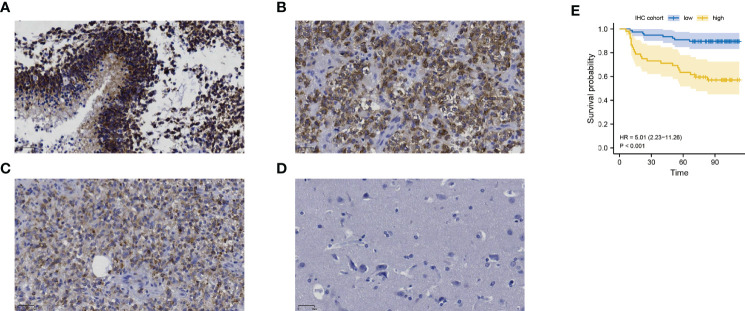
The expression of DDOST in gliomas and its prognostic significance were analyzed by immunohistochemistry. **(A–C)** showed that DDOST is strongly, moderately, and weakly positive in gliomas, respectively. **(D)** shows the expression of DDOST in normal brain tissue. High DDOST expression in glioma was related to poor OS **(E)**.

We further performed an expression analysis about DDOST in gliomas using sequencing analysis data from CGGA, GEO, TCGA, and GTEx databases. Results showed that DDOST expression was significantly higher than that in normal brain tissues (**Figures 2A–E**). In addition, the relationship between DDOST expression and different clinical features was also analyzed. Significantly higher expression of DDOST was identified in those clinical features known to confer worse prognosis, such as age ≥ 42, high grade, IDH wild type, 1p19q non-codel, and MGMT methylation (**Figure 3**). In the CGGA 693 cohort, which was used as an external validation dataset, we reached the same conclusion (**Supplementary 2**). Next, we investigated the impact of DDOST on survival of gliomas using the CGGA 325 and CGGA 693 datasets. The high/low expression of DDOST was determined by the median value. Kaplan–Meier survival analysis revealed that patients with high DDOST expression had significantly worse OS than those with low expression (HR = 5.11, 95% CI: 3.76–6.95, *p* ≤ 0.001, **Figure 4A**). Stratification analysis was carried out on DDOST to assess its impact on glioma prognosis. Patients with glioma were divided into different groups based on different clinical characteristics for age (age<42, age≥42), gender (female, male), grade (WHO 2, WHO 3, and WHO 4), IDH (mutant, wild type), 1p19q (codel, non-codel), and MGMT (methylated, un-methylated) status. Survival analysis showed that patients with high expression of DDOST had a poor prognosis in various subgroups (**Figures 4B–N**). However, in the 1p19q codel group, we observed only patients with low expression of DDOST. We obtained similar results from the external validation set (**Supplementary 3**). Immunohistochemistry of tissue microarray was used to analyze and confirm the relationship between DDOST expression and prognosis. As shown in **Figures 5A–C**, DDOST expression was significantly positively correlated with WHO grade, with a strong positivity in WHO 4, a moderate positivity in WHO 3, and a weak positivity in WHO 2. Brain tissue from normal individuals had no evidence of DDOST expression (**Figure 5D**). Survival analysis showed that the OS was significantly poorer in cases with high DDOST expression (HR = 5.01, 95% CI: 2.23–11.26, *p* < 0.001, **Figure 5E**). All of the above results strongly suggest that DDOST can accurately predict the prognosis of glioma patients.

### DDOST Was Heterogenous in the Tumor Immune Microenvironment

**Figure 6:**
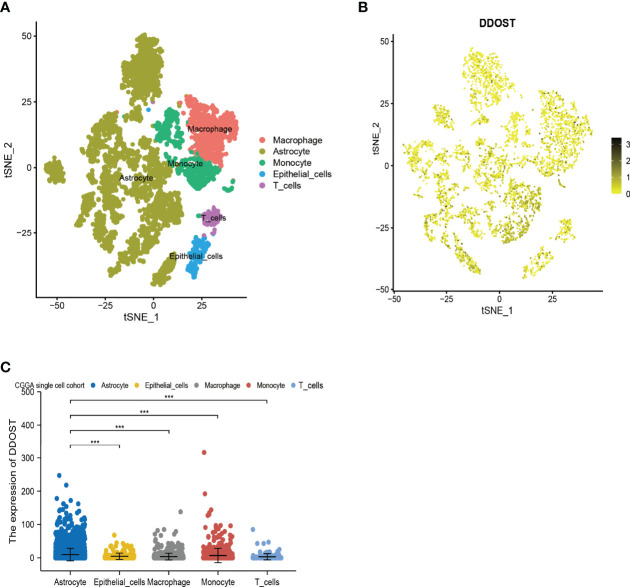
The expression of DDOST in glioma by single-cell analysis. **(A)** By dimensionality reduction analysis of CGGA single-cell data, 6,148 cells were divided into astrocytes, epithelial cells, macrophages, monocytes, and T cells. **(B)** Scatter plot of DDOST distribution in glioma. **(C)** The scatter plot shows the expression of DDOST in astrocytes, epithelial cells, macrophages, monocytes, and T cells. ***p < 0.001.

In 6,148 cells from 13 gliomas, cells were mainly divided into 5 types, namely, astrocytes, macrophages, epithelial cells, monocytes, and T cells (**Figure 6A**). Based on single-cell analysis, the expression of DDOST was significantly different in different clusters (**Figure 6B**). DDOST expression was increased in some clusters, especially in astrocytes, macrophages, and monocytes (**Figure 6C**).

### DDOST Was Associated With Cancer-Promoting Pathways

**Figure 7:**
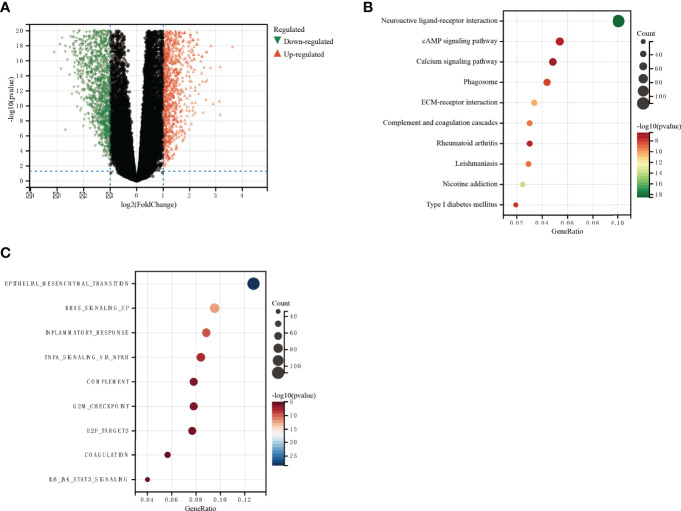
GO and KEGG functional enrichment analysis of the role of DDOST in glioma. **(A)** The volcano map shows the differential genes between high- and low-DDOST groups by the CGGA 325 cohort **(A)**. KEGG **(B)** and GO **(C)** were used to analyze the relevant mechanisms.

There were 1,279 DEGs, of which 613 were upregulated and 666 were downregulated (**Figure 7A** and **Supplementary 4**). DAVID analysis was conducted on the above differential genes to identify functional enrichments. The results showed that the differential genes were significantly enriched in neuroactive ligand–receptor interaction, cAMP signaling pathway, calcium signaling pathway, epithelial–mesenchymal transition, KRAS, inflammatory response, and other pathways (**Figures 7B, C**). Enrichment results of GSEA did not depend on differential genes, and their results together with those of DAVID could better predict the underlying mechanisms of DDOST. Our GSEA analysis identified pathways associated with glucose metabolism, and oncogenic and tumor microenvironment (TME) as being enriched (**Supplementary Figures 3A, B**).

### The Expression of DDOST Was Related to the Infiltration of Immune Cells in the Tumor Microenvironment of Gliomas

**Figure 8:**
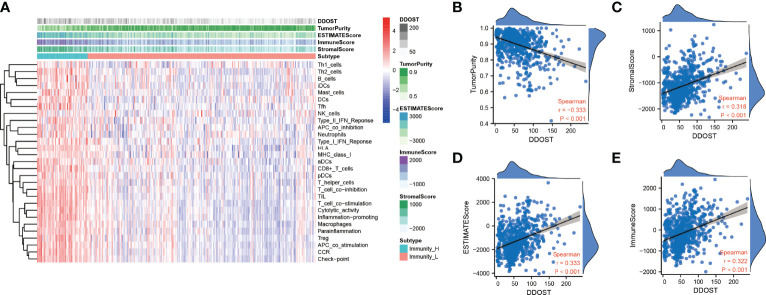
Immune infiltration patterns of low- and high-DDOST analyzed by ssGSEA methods in glioma from the CGGA dataset. **(A)** Heatmap revealing the scores of immune cells in low and high immunities. **(B–E)** Scatter plot showing the correlation between DDOST and tumor purity, stromal, ESTIMATE, and immune scores.

**Figure 9:**
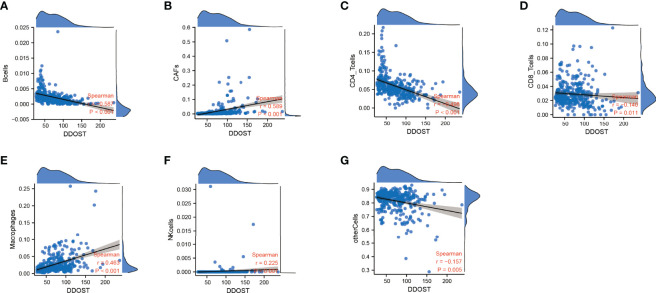
The relationship between the expression of DDOST and immune cells were analyzed by EPIC. The scatter plot shows the correlation between DDOST and B cells **(A)**, CAFs **(B)**, CD4+ T cells **(C)**, CD8+ T cells **(D)**, macrophages **(E)**, NK cells **(F)**, and other cells **(G)**.

**Figure 10:**
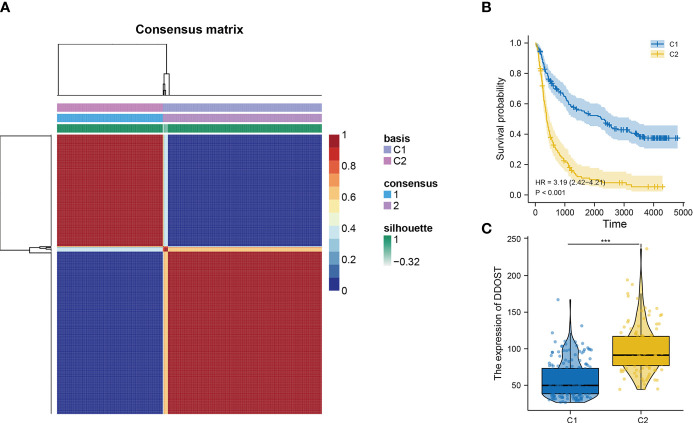
The relationship between DDOST and tumor immune infiltration was analyzed by NMF cluster analysis of glioma patients with immune-related genes. **(A)** According to the cophenetic value and the expression of immune-related genes, glioma patients were divided into two clusters, C1 and C2. **(B)** The survival curve was used to analyze the survival difference between C1 and C2 groups. **(C)** The expression differences of DDOST in C1 and C2 clusters were compared. ***p < 0.001.

The infiltration of immune cells was assessed using ssGSEA. The immune cell infiltration status of the patients was used to categorize them into low and high immunity groups. Results showed that the expression of DDOST in the group with more immune cell infiltration was significantly higher than that with lower immune cell infiltration (**Figure 8A**). The expression level of DDOST was negatively correlated with tumor purity (*r* = −0.333, *p* < 0.001, **Figure 8B**). However, it was positively correlated with stromal score (*r* = 0.318, *p* < 0.001, **Figure 8C**), ESTIMATE score (*r* = 0.333, *p* < 0.001, **Figure 8D**), and immune score (*r* = 0.322, *p* < 0.001, **Figure 8E**). Furthermore, we analyzed the relationship between DDOST and infiltrating B cells, CAFs, CD4+ T cells, CD8+ T cells, macrophages, NK cells, and other cells through EPIC. The expression of DDOST was negatively correlated with the infiltrating B cells (r = -0.587, p < 0.001) and CD4+ T cells (r = -0.498, *p* < 0.001). However, it was positively correlated with the infiltrating CAFs (r = 0.589, *p* < 0.001) and macrophages (r = 0.463, *p* < 0.001). However, there was no significant correlation between the expression of DDOST and NK cells and other cells. Scatter plots of the association of DDOST with these immune cells are shown in **Figures 9A–G**. Then, we obtained 2,483 immune-related genes from the website (**Supplementary Table 3**). NMF clustering analysis was performed according to the expression of these genes. According to the values of the cophenetic, we clustered gliomas into 2 categories, Cluster 1 and Cluster 2 (**Figure 10A**). Survival analysis showed that patients in group C2 had a significantly worse survival than those in group C1 (**Figure 10B**). Interestingly, we also found that the expression of DDOST was significantly higher in group C2 than in group C1 (**Figure 10C**).

### Different DDOST Expression Exhibited Distinct Molecular Features

We detected 509 and 153 samples containing mutations in LGG and GBM. According to the expression of DDOST, gliomas were divided into high- and low-expression groups. Waterfall plots show the differences of the first 15 mutation genes. We found that IDH mutations exist in 80.6% of LGG, and high expression of DDOST had less IDH mutation. However, gene alteration of CIC and FUBP1 in the low-expression DDOST group was significantly higher than that in the high-expression group (**Supplementary Figure 4A**). In GBM, TP53 had the highest rate of gene mutation, but the difference between the two groups was not significant. In the low-expression group, the mutation rate of PTEN was higher (**Supplementary Figure 4B**).

### DDOST Could Better Improve the Predictive Model

**Figure 11:**
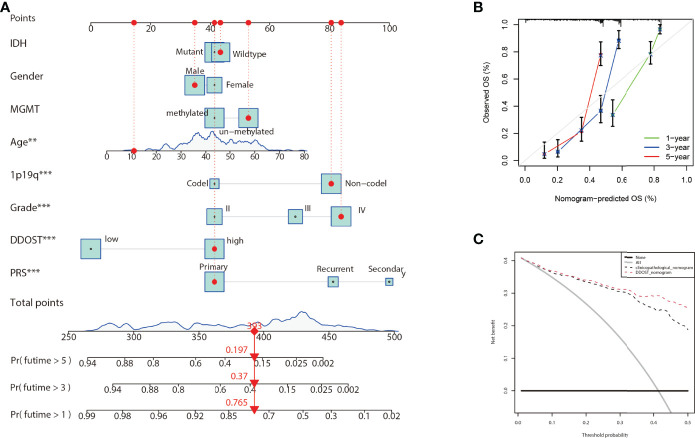
Nomogram for the prediction of prognostic probabilities in the CGGA dataset. **(A)** The nomogram for the prediction of OS was developed using the CGGA dataset. **(B)** The calibration plots for predicting 1-, 3-, and 5-year survival. **(C)** Decision curve analysis for the DDOST nomogram and the clinicopathological nomogram to estimate the OS.

Nomograms are powerful tools that can be used to determine individuals’ risk in a clinical setting based on the integration of multiple factors (17). A nomogram incorporating DNA repair genes, age, PRS, grade, IDH, 1p19q, and MGMT status was created to predict the probability of 1-, 3-, and 5-year OS. As illustrated in **Figure 11A**, a score was assigned to each of the above features. We predicted patient 1-, 3-, and 5-year survival by calculating the sum of the scores for each glioma patient. For example, a patient with an overall score of 393 would have a 5-year survival of 19.7%, a 3-year survival of 37%, and a 1-year survival of 76.5%. According to calibration curves, actual and predicted survival were very close (**Figure 11B**). The DCA curve showed that DDOST combined with other clinical traits can bring benefits to predict the prognosis of glioma patients (**Figure 11C**).

## Discussion

Malignancies of the brain account for 1.6% of new cancers and 2.5% of deaths annually worldwide (18). Gliomas account for 80% of brain malignancies. To date, surgery remains to be the first-line treatment for gliomas, and maximal safe resection is the mainstay and the most effective therapeutic option. Conventional radiotherapy combined with chemotherapy based on temozolomide and PCV (procarbazine, lomustine, and vincristine) after maximal safe resection is the standard adjuvant treatment in high-grade glioma and some WHO 2 gliomas, and tumor treating fields (TTFields) are newly recommended for glioblastoma according to the 2021 NCCN guideline (https://www.nccn.org/professionals/physician_gls/pdf/cns.pdf). Unfortunately, the prognosis is still unsatisfactory, especially for glioblastoma, which has a 5-year survival rate of 3%. Evidence shows that genetic alterations are involved in the development of gliomas (3). The leading change in the 2021 fifth edition WHO classification is the recommendation of molecular diagnostics, which could provide the most accurate classification of CNS neoplasms and guide clinical management and prognosis of gliomas. For example, the presence of combined 1p/19q loss and/or IDH1 mutations predicts a favorable prognosis, and could get benefit from PCV chemotherapy.

A glycosylated protein is an important form of protein modification in eukaryotes, occurring in more than 50% of all proteins (19). Moreover, evidence shows that the glycosylation of proteins is closely related to the biological behavior of tumors. In particular, OST-mediated protein glycosylation is a key factor of signal transduction, protein folding, and degradation. Some studies have also shown that it is closely related to the immune escape of tumor cells (20). A subunit of the OST complex, DDOST, plays a vital role in N-glycosylation (21). It has been shown that DDOST also plays an important role in tumors and can serve as a marker for HCC and is closely associated with the TME (7). Numerous studies on glycomics and glycoproteomics have established a strong link between glycosylation and gliomas (22, 23). In this study, we firstly analyzed the expression and significance of DDOST in pan cancer. Results showed that DDOST expression was upregulated in most tumors compared with the corresponding normal tissues including gliomas, and tumors that expressed high levels of DDOST displayed poor prognosis. IHC analysis of tissue microarray confirmed our data, and DDOST expression was significantly positively correlated with WHO grade, with a strong positivity in WHO 4, a moderate positivity in WHO 3, and a weak positivity in WHO 2, and negative in brain tissue from normal individuals. We further found that DDOST was overexpressed in patients with worse molecular features, which predicted poor survival, such as IDH wild type, 1p19q non-codel, and MGMT un-methylated. Patients with high expression of DDOST had worse prognosis than those with lower levels in gliomas. These results were in accordance with a previous study by Shapanis et al., who discovered that in cervical and oropharyngeal cancer, an increased expression of ANXA5 and DDOST was associated with a shorter time to metastasis and decreased survival (6). It suggests that DDOST may be a candidate oncogene and an independent factor for predicting poor prognosis in gliomas.

It is widely known that tumors consist not only of cancerous cells but also of non-cancerous cells containing a significantly altered surrounding stroma including immune cells, endothelial cells, fibroblasts, and molecules produced and released by them, which is called the TME. Evidence shows that the tumor cells could interact with TME by releasing extracellular signals, promoting tumor angiogenesis, and inducing immunity tolerance to influence tumorigenesis, tumor progression, therapeutic response, and clinical outcome (24). Now, TME has become a therapeutic target in cancers. For example, anti-VEGF and EGFR agents could induce tumor vascular normalization, and immunotherapy drugs including anti-PD-1 (programmed death-1) and anti-PD-L1 could remodel the immuno-microenvironment by regulating the immune cells to treat tumors (25).

Gliomas are a group of immunosuppressive tumors with a complicated TME. Although immunotherapy has achieved remarkable survival benefits in multiple cancers, due to the blood–brain barrier (BBB), the effectiveness of target and immunotherapy drugs is dramatically reduced in gliomas. Furthermore, gliomas have a high heterogeneity, and show differences in grade of malignancy and molecule characteristics. Researching the molecular biomarkers and unique immunological status of gliomas has a profound effect on cancer diagnosis and therapeutic strategies. New studies have found that some biomarkers, such as ITGA-5 and Tenascin-C, could predict glioma prognosis, and are closely related to the immunosuppressive microenvironment, particularly for the clinical application of immunotherapy in glioma (26, 27). New studies also reported that DDOST could interact with the microenvironment of gliomas and was negatively related to tumor-infiltrating B cells and CD4+ T cells and positively related to CAFs and tumor-associated macrophages (TAMs). B lymphocytes form the major immune cell population in the TME and contribute to tumor progression (28). In recent studies, however, it has been found that the density of B and Th1 cells was closely correlated to improving patient survival (29). Our data showed that higher levels of DDOST expression were associated with fewer tumor-infiltrating B cells and CD4+ T cells in glioma, which meant that less tumor-infiltrating B cells were unable to activate a powerful antitumor immune response and induce immune escape, which, in turn, led to a poorer prognosis. CD4+ T cells play an anti-tumor role in the TME. Our results showed that DDOST expression was inversely correlated with CD4+ T cells and further illustrated that DDOST may contribute to the immunosuppressive microenvironment of gliomas, which affected the effective of immunotherapy drugs. Many studies have demonstrated that CAFs are not individual cells that surround tumors, but rather interact with cancer cells to promote tumor growth and survival and maintain their malignant propensity (30). TAMs are a key component of the cancer microenvironment, and contribute to tumor growth and progression (31). Based on our immunoinfiltration analysis, we concluded that DDOST was associated with an immunosuppressive microenvironment in gliomas. Lastly, based on the above data, we constructed a nomogram incorporating DNA repair genes, age, PRS, grade, IDH, 1p19q, and MGMT status to predict the probability of 1-, 3-, and 5-year OS. One-, 3-, and 5-year OS could be predicted by calculating the sum of the scores for each glioma patient. For example, a patient with an overall score of 393 would have a 5-year survival of 19.7%, a 3-year survival of 37%, and a 1-year survival of 76.5%. The DCA curve showed that DDOST combined with other clinical traits can bring benefits to predict the prognosis of glioma patients.

These results were mainly based on the data of bioinformatics; therefore, large-sample immunohistochemistry data, Western blot, and RT-PCR of glioma tissues are urgently needed to confirm the above results. We will also further detect and confirm these results in *in vivo* and *in vitro* experiments in glioma.

In the present study, we found that DDOST could be used as an accurate predictor for patients with gliomas. The immune infiltration analysis showed that DDOST was closely related to the TME and might mediate the suppressive immune microenvironment of glioma. Finally, DDOST and clinical features were used to construct a nomogram that could accurately assess the prognosis of patients. Our results preliminarily suggest that DDOST could serve as a potential target for the treatment of glioma patients, and an independent factor for predicting prognosis in gliomas, but further detailed mechanisms need to be explored in future research.

## Data Availability Statement

The datasets presented in this study can be found in online repositories. The names of the repository/repositories and accession number(s) can be found in the article/**Supplementary Material**.

## Ethics Statement

Ethical review and approval were not required for the study on human participants in accordance with the local legislation and institutional requirements. Written informed consent for participation was not required for this study in accordance with the national legislation and the institutional requirements.

## Author Contributions

YY and GW were responsible for the overall design of this study. XC and RZ were responsible for manuscript writing as well as data analysis. JP mainly performed immunohistochemistry and scoring. TY and XW were responsible for data collection and R code writing. CG assisted GW with manuscript writing. All authors contributed to the article and approved the submitted version.

## Funding

This study was supported by the Special pharmaceutical research project of Hebei Pharmaceutical Association Hospital (2HC2022012).

## Conflict of Interest

The authors declare that the research was conducted in the absence of any commercial or financial relationships that could be construed as a potential conflict of interest.

## Publisher’s Note

All claims expressed in this article are solely those of the authors and do not necessarily represent those of their affiliated organizations, or those of the publisher, the editors and the reviewers. Any product that may be evaluated in this article, or claim that may be made by its manufacturer, is not guaranteed or endorsed by the publisher.

## Supplementary Material

Supplementary Figure 1 Expression difference of DDOST between different clinical characters in patients with glioma in the CGGA 693 cohort. The expression of DDOST in different age **(A)**, gender **(B)**, PRS type **(C)**, grade **(D)**, IDH **(E)**, 1p19q **(F)**, and MGMT status **(G)**.

Supplementary Figure 2 Prediction of outcome of the DDOST in stratified patients in the CGGA 693 dataset. Survival curve was used to analyze OS in the low- and high-DDOST groups in CGGA 325 set(A). Survival analysis of the signature in patients stratified by age **(B, C)**, gender **(D, E)**, grade **(F–H)**, IDH **(I, J)**, 1p19q status** (K, L)**, and MGMT promoter **(M, N)**.

Supplementary Figure 3 The role of DDOST in glioma was analyzed by GSEA. GO**(A)** and hallmark**(B)** gene sets were performed to explore the mechanism of DDOST in glioma.

Supplementary Figure 4 Waterfall diagram shows the relationship between DDOST expression and gene mutation in LGG **(A)** and GBM **(B)**.

Supplementary Table 1 Clinicopathological characteristics of glioma patients from the GEO, TCGA, CGGA database and tissue microarray.

Supplementary Table 2 The differential genes between high- and low-DDOST groups by CGGA 325 cohort.

Supplementary Table 3 Immune-related genes.
