# Supplementary figures and images for: DDOST Correlated with Malignancies and Immune Microenvironment in Gliomas
Source: Front Immunol. 2022 Jun 23;13:917014. doi: 10.3389/fimmu.2022.917014 (PMC9260604; doi:10.3389/fimmu.2022.917014)

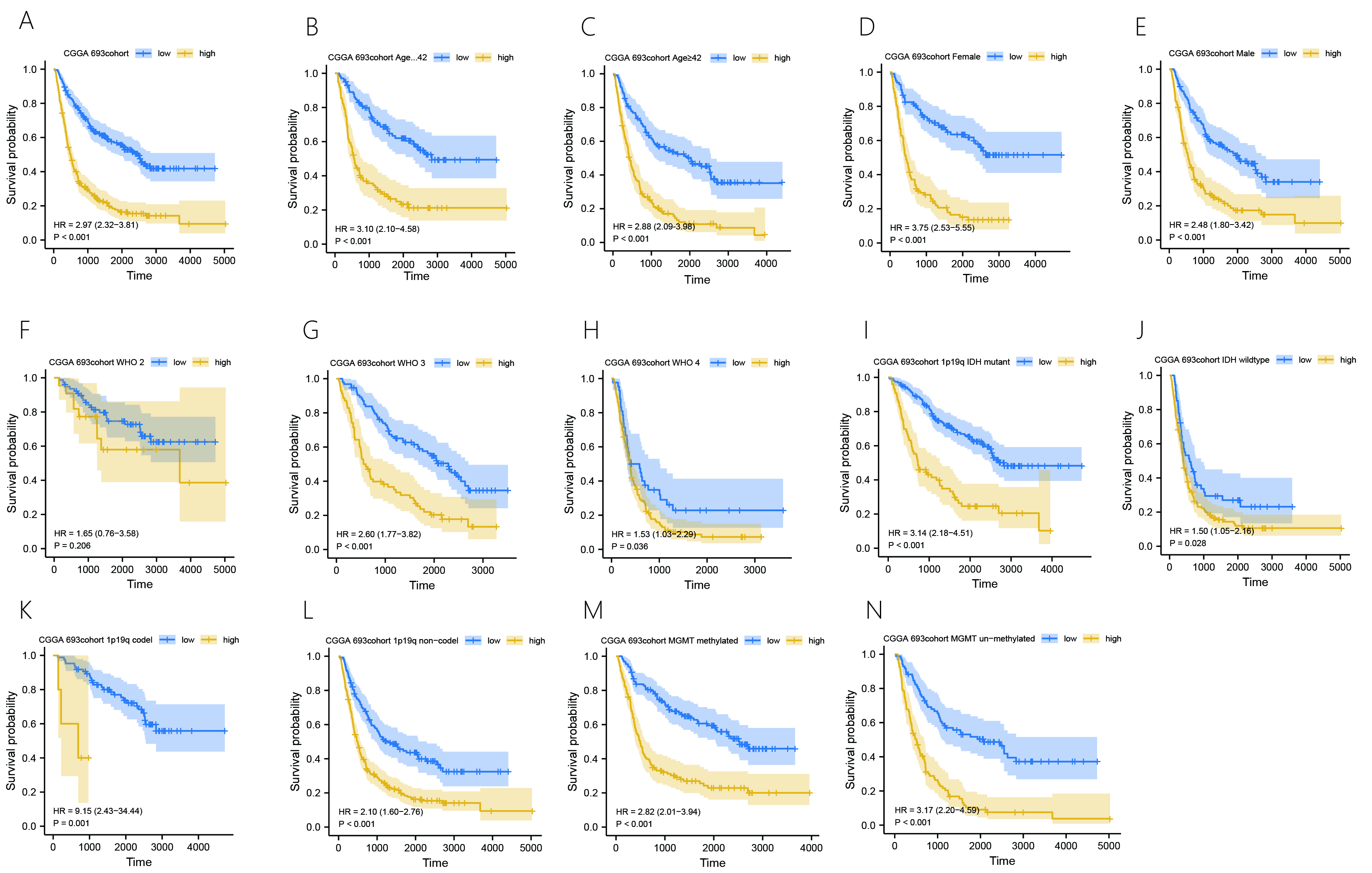

Supplement: Supplementary Figure 1 — Expression difference of DDOST between different clinical characters in patients with glioma in the CGGA 693 cohort. The expression of DDOST in different age (A), gender (B), PRS type (C), grade (D), IDH (E), 1p19q (F), and MGMT status (G). [file Image_1.tif]

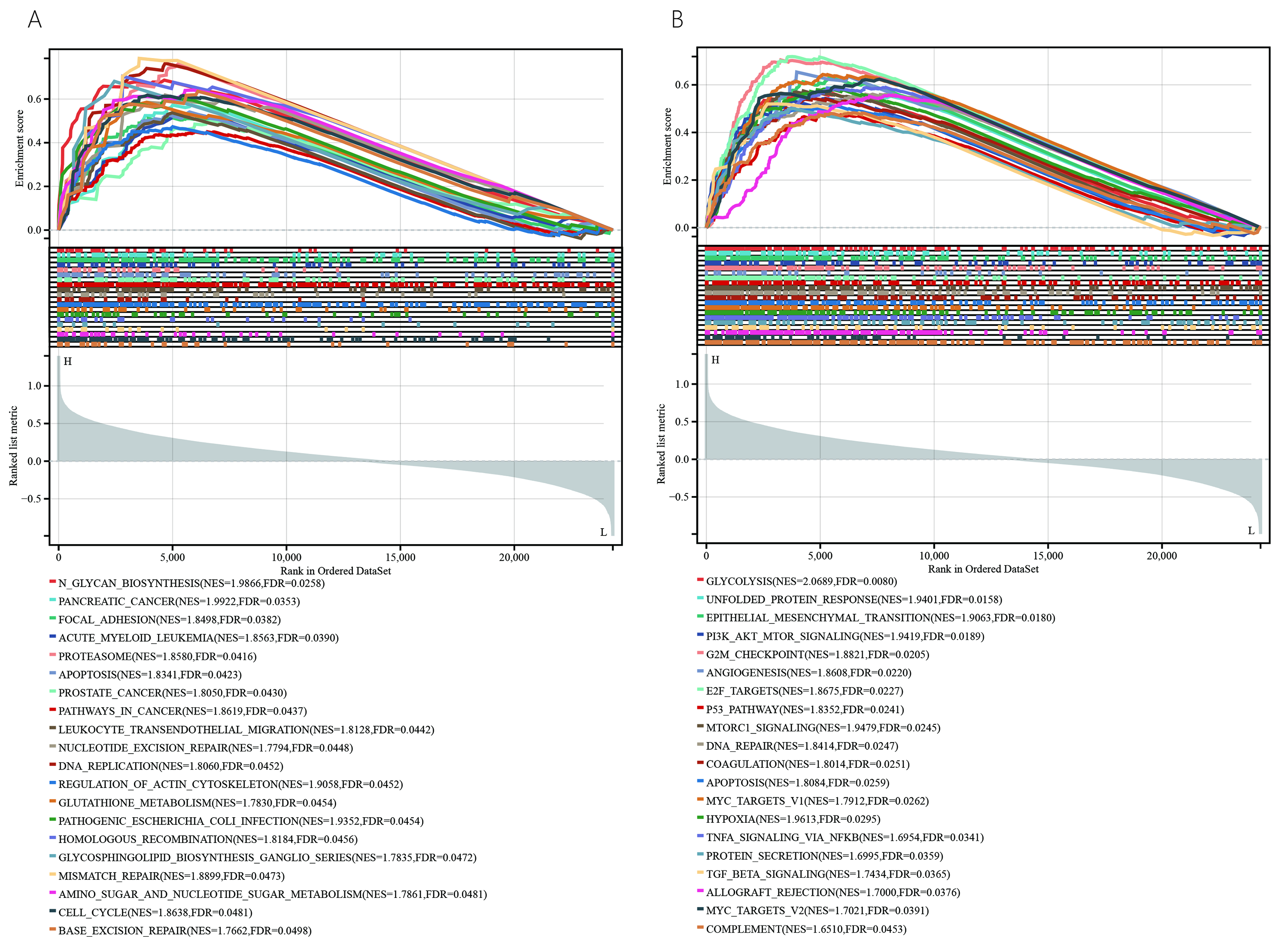

Supplement: Supplementary Figure 2 — Prediction of outcome of the DDOST in stratified patients in the CGGA 693 dataset. Survival curve was used to analyze OS in the low- and high-DDOST groups in CGGA 325 set(A). Survival analysis of the signature in patients stratified by age (B, C), gender (D, E), grade (F–H), IDH (I, J), 1p19q status (K, L), and MGMT promoter (M, N). [file Image_2.tif]

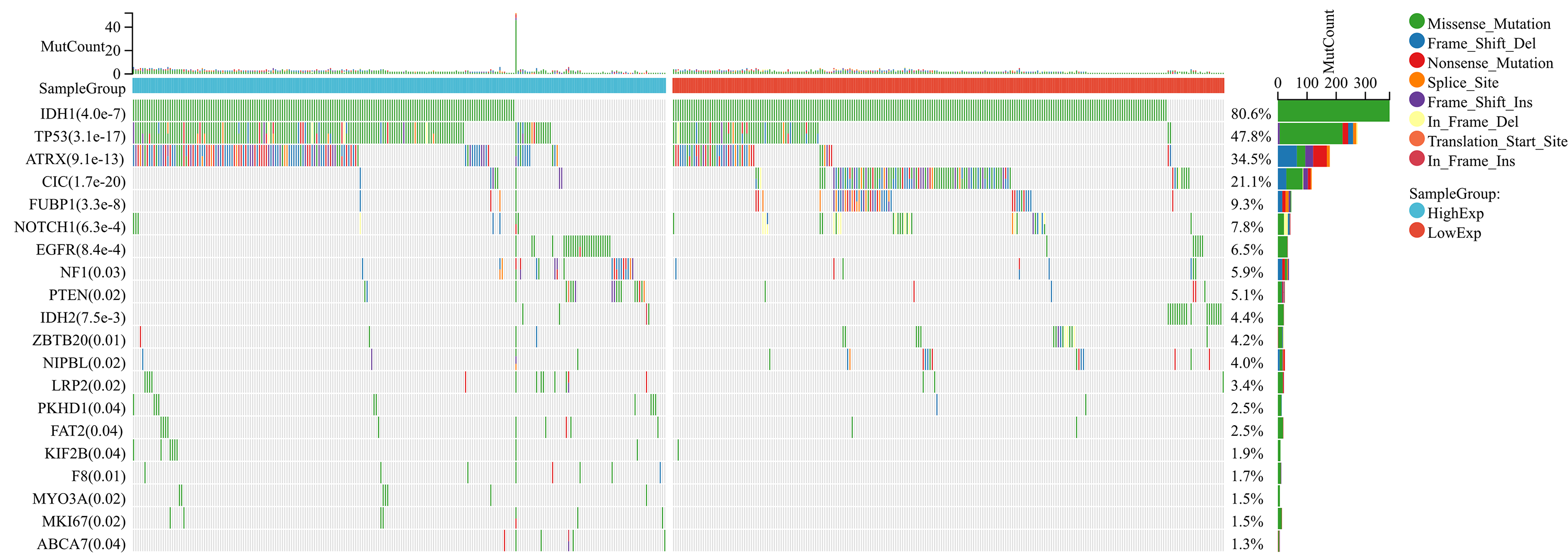

Supplement: Supplementary Figure 3 — The role of DDOST in glioma was analyzed by GSEA. GO(A) and hallmark(B) gene sets were performed to explore the mechanism of DDOST in glioma. [file Image_3.tif]

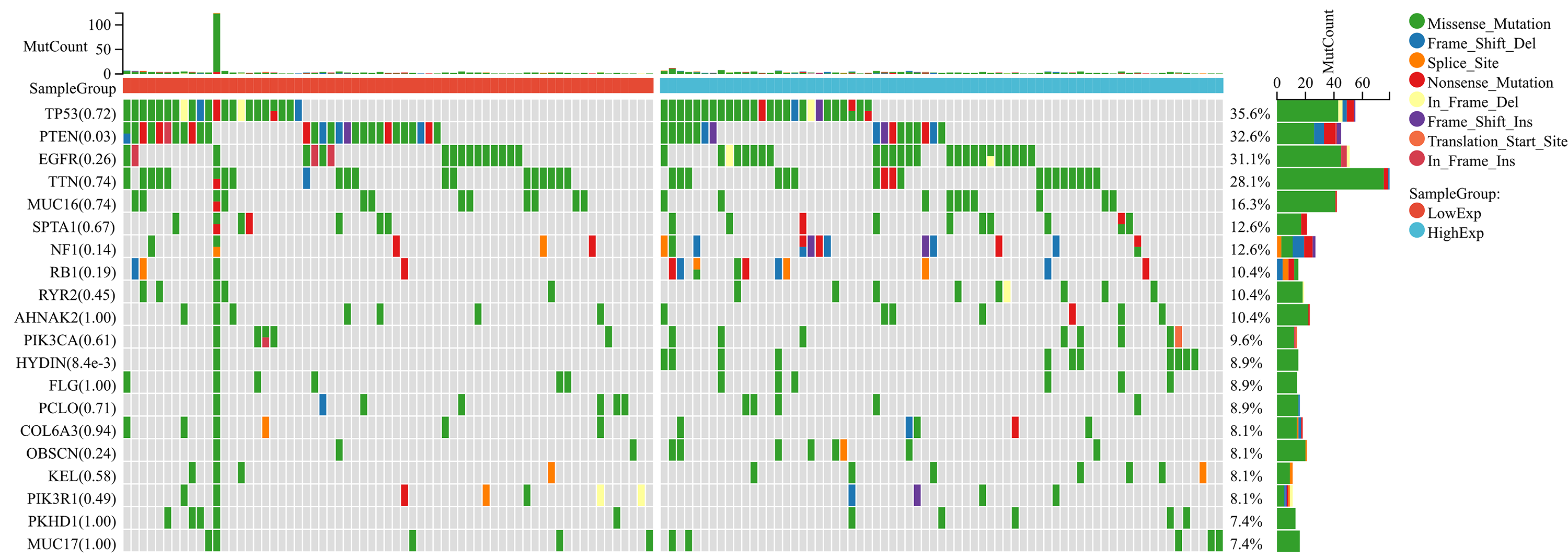

Supplement: Supplementary Figure 4 — Waterfall diagram shows the relationship between DDOST expression and gene mutation in LGG (A) and GBM (B). [file Image_4.tif]
