# Supplementary material for: DDOST Correlated with Malignancies and Immune Microenvironment in Gliomas
Source: Front Immunol. 2022 Jun 23;13:917014. doi: 10.3389/fimmu.2022.917014 (PMC9260604; doi:10.3389/fimmu.2022.917014)
Supplement: Supplementary Table 1 — Clinicopathological characteristics of glioma patients from the GEO, TCGA, CGGA database and tissue microarray. [file Table_1.docx]

Table1 Clinicopathological characteristics of glioma patients from the GEO, TCGA, CGGA database and tissue microarray.

|  | GSE4290  (n=176) | GSE50161  (n=130) | TCGA  (n=592) | CGGA325  (n=286) | CGGA693  (n=429) | Tissue Microarray  (n=124) |
| --- | --- | --- | --- | --- | --- | --- |
| Age |  |  |  |  |  |  |
| ＜42 | NA | NA | 241 | 124 | 197 | 44 |
| ≥42 | NA | NA | 346 | 162 | 232 | 77 |
| Gender |  |  |  |  |  |  |
| Female | NA | NA | 246 | 110 | 191 | 35 |
| Male | NA | NA | 341 | 176 | 238 | 86 |
| Normal Tissue | 23 | 13 | 5 | NA | NA | 3 |
| Tumor Tissue | 153 | 117 | 587 | 286 | 429 | 121 |
| Grade |  |  |  |  |  |  |
| II | 45 | NA | 211 | 86 | 100 | 37 |
| III | 31 | NA | 234 | 68 | 164 | 40 |
| IV | 77 | NA | 142 | 132 | 165 | 47 |
| IDH Status |  |  |  |  |  |  |
| Wildtype | NA | NA | 219 | 136 | 193 | NA |
| Mutation | NA | NA | 368 | 150 | 236 | NA |
| 1p/19q |  |  |  |  |  |  |
| Codel | NA | NA | 149 | 57 | 90 | NA |
| Non-codel | NA | NA | 438 | 229 | 339 | NA |
| MGMT |  |  |  |  |  |  |
| Methylated | NA | NA | NA | 147 | 251 | NA |
| Un-methylated | NA | NA | NA | 139 | 178 | NA |
| Status |  |  |  |  |  |  |
| Dead | NA | NA | 173 | 202 | 278 | 47 |
| Alive | NA | NA | 414 | 84 | 151 | 77 |
